# Intention to Receive COVID‐19 Vaccination and Its Associated Factors Among Community in Motta Town, North West Ethiopia, 2022: Application of Theory of Planned Behavior

**DOI:** 10.1002/puh2.70383

**Published:** 2026-09-23

**Authors:** Alemnew Getachew, Yosef Wasihun, Nestanet Fentahun, Gebeyehu Lakew

**Affiliations:** ^1^ Motta District Health office, Amhara Region Bahir Dar Ethiopia; ^2^ Department of Nutrition Bahir Dar University College of Medicine and Health Science Bahir Dar Ethiopia; ^3^ Department of Health Promotion and Health Behavior School of Public Health College of Medicine and Health Sciences University of Gondar Gondar Ethiopia

**Keywords:** COVID 19 vaccination, Ethiopia, intention, theory of planned behavior

## Abstract

**Background:**

The coronavirus disease‐19 (COVID‐19) pandemic has significantly impacted global health, with vaccine hesitancy emerging as a major obstacle to controlling the disease. Despite the availability of effective vaccines, many individuals remain reluctant to receive them. This study aims to investigate the factors influencing COVID‐19 vaccine intention among the community in Motta town, Ethiopia, using the theory of planned behavior.

**Objectives:**

To assess intention to receive COVID‐19 vaccine and associated factors among the community in Motta town, North West Ethiopia.

**Method:**

A community‐based cross‐sectional study was conducted among 630 unvaccinated adults in Motta town, North West Ethiopia, from May 15 to June 15, 2022. A systematic random sampling technique was used to select study participants. Data were collected by using interviewer‐administered questionnaires and analyzed using descriptive statistics and multiple linear regression. In the final model, a confidence interval (95%) and a *p* value <0.05 were considered statistically significant.

**Result:**

Overall, 310 (49.2%; 95% CI: 45.2%–53.0%) participants intended to receive the COVID‐19 vaccine. The mean score of intention was 12.38 with SD ± 5.300. Religion (Muslim) (*B* = 0.862, *p* < 0.004), educational status (no formal education) (*B* = −1.065, *p* < 0.029), primary school education (*B* = −0.879, *p* < 0.038), attitude (*B* = 0.170, *p* < 0.000), subjective norm (*B* = 0.027, *p* < 0.001), and perceived behavioral control (PBC) (*B* = 0.029, *p* < 0.004) were significant factors associated with vaccination intention.

**Conclusion:**

Approximately half of the unvaccinated community members intended to receive the COVID‐19 vaccine. Religion, educational status, attitude, subjective norm, and PBC were significant predictors of vaccination intention. Public health interventions should focus on improving positive attitudes, strengthening community support for vaccination, enhancing individuals’ confidence in accessing vaccination, and addressing access‐related barriers to increase COVID‐19 vaccine uptake.

## Graphical abstract

Among 630 unvaccinated adults in Motta Town, Ethiopia, only 49.2% intended to receive the COVID‐19 vaccine. Religion, educational status, attitude, subjective norm, and perceived behavioral control significantly predicted vaccination intention, highlighting the need for behavioral and structural interventions to improve vaccine uptake.

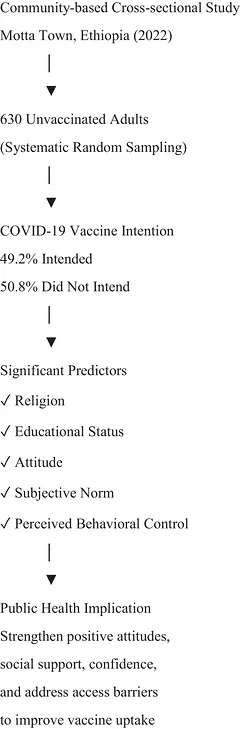

## 1 Background

In late 2019, a unique case of pneumonia that resulted in death was discovered in Wuhan, China. The cause of the disease was quickly recognized as a previously unknown beta coronavirus, which was given the designation severe acute respiratory syndrome coronavirus 2 (SARS‐CoV‐2) by the World Health Organization (WHO) [1].

The SARS‐CoV‐2 causes coronavirus disease‐19 (COVID‐19), which is highly contagious, and when someone infected with this virus sneezes or coughs, small droplets from their nose or mouth can easily spread the infection. However, it has a tremendous impact on the psychosocial well‐being, both socially and economically, of the population [2].

On March 11, 2020, the WHO declared COVID‐19 to be a worldwide pandemic [3]. As the Worldometer report showed, until April 6, 2022, 494,627,998 cases and 6,185,161 deaths were recorded in 225 countries. With 181,875,160 cases and 1,780,362 deaths, Europeans are the most affected. The United States has the highest number of cases worldwide (81,906,213) and the highest number of deaths (1,009,462), whereas India has the second highest number of cases (43,030,925) and deaths (521,518). Highly affected one is Brazil's case (30,040,129) and death (660,586). The African region has so far been the least affected, with a total of 11,784,863 and 100,067 deaths. However, the statistics keep going up. In Ethiopia, we have confirmed 469,879 cases and 7508 deaths, and in the Amhara region, we have confirmed 11,791 cases and 293 deaths [4, 5].

The coronavirus pandemic's rapid spread has put a large burden on almost the entire population of the United States. The European zone had a 98% mortality rate, whereas Africa had a 10% mortality rate. Coronavirus still produces significant morbidity and mortality all over the world, with an impact of 41% in Africa. South Africa has 24%, Egypt has 4%, Nigeria has 4%, Algeria has 6%, and the rest of the world has 24%. Only five African countries are responsible for 75% of all cases and 76% of all deaths [6].

The coronavirus has quickly escalated into a serious public health disaster. The majority of deaths and serious consequences occurred in vulnerable groups such as elderly people, pregnant women, and healthcare providers; hence, SARS‐CoV‐2 vaccination was recommended first in these groups [7].

Successful vaccines emerged as vital to disease and death by COVID‐19 virus, as individuals who were not fully vaccinated 10 times to be hospitalized and 11 times died, and Moderna, Pfizer, and Johnson and Johnson were all 86% effective in preventing hospitalization [8, 9].

The WHO says fighting against vaccine‐preventable diseases is a major problem, and most countries have begun vaccination campaigns [10]. Until March 5, 2022, at least one dose of the COVID‐19 vaccine has been given to 63.1% of the world's population. Globally, 10.83 billion doses have been administered, with 22.65 million doses being administered every day. In low‐income countries, only 13% of the population got at least one dose [11].

One of the primary obstacles to managing the COVID‐19 pandemic is the refusal of some people to take the COVID‐19 vaccine [12]. In Indonesia and China, there has been a significant rate of vaccine reluctance. The top reasons for uncertainty were vaccine efficacy and safety [13, 14]. More than half of Ethiopians ready for the COVID‐19 vaccine had a definite intent and were unsure (32.8%) or unwilling (20.7%) to get vaccinated. This falls short of the WHO's objective of vaccinating 70% of the population to achieve herd immunity [15].

The uptake of any COVID‐19 vaccination is a significant obstacle. More than a third of the population said that they were undecided or did not plan to get the vaccine. Refusing to get vaccinated affects or may affect a vast number of individuals, with significant social, economic, and health consequences. Receiving the COVID‐19 vaccine by the general public is the first step in achieving a high rate of COVID‐19 vaccine uptake and controlling the COVID‐19 pandemic [16].

Despite the availability of COVID‐19 vaccines, some members of the public are unlikely to get vaccinated. As a result, it is critical to understand the intentions, motivators, and barriers that influence the general public's decision to vaccinate against COVID‐19. The knowledge would aid in the development of intervention strategies that are accessible to the broader public while also targeting populations that have a low vaccination rate [17].

This study will employ the theory of planned behavior (TPB) to understand why people do not receive the COVID‐19 vaccine and identify factors that can serve to support community attitudes, knowledge, intentions, and environmental constraints toward receiving the COVID‐19 vaccine. TPB was developed by Fishbein and Ajzen in 1970s. The model assumed that the best predictor of a behavior is behavioral intention, which, in turn, determined by attitude toward the behavior, subjective normative, and perceived behavioral control (PBC) of performing the behavior. TPB has been used successfully to predict and explain a wide range of health behaviors and intentions, including health services utilization like immunization services [18, 19].

The TPB is an expansion of the theory of reasoned action, and adding PBC constructs an independent determinant of behavior intention. So this model is important for this study because it takes into account factors outside individual control that may affect intention to receiving COVID‐19 vaccine [18, 19].

The previous study was an online survey in which those who were uneducated and did not have a smartphone did not participate, and existing Ethiopian research was facility‐based and focused on the literate groups (health personnel and teachers) rather than the entire community [20]. This study was community‐based and was explore factors that influence people's preparedness to be vaccinated in order to design effective interventions to improve the widely available COVID‐19 vaccine. Behavioral intention to receive the COVID‐19 vaccine is crucial for the implementation of intervention. There is currently limited research in Ethiopia regarding the intention of receiving COVID‐19 amongst the community, specifically in the study area. Therefore, this study aimed to assess the intention to receive the COVID‐19 vaccine among the community in Motta town, Ethiopia.

## 2 Methods and Materials

### 2.1 Study Setting and Period

The study was conducted in Motta town, Amhara Region, North West Ethiopia. It is located in East Gojjam, 368 km north of Addis Ababa and 120 km away from Bahir Dar. There is one general hospital, one health center, and six health posts in the town. There are also six kebeles (01–06) in the town. Currently, the total population of the town is 54,964; males are 27,427, and females are 27,537. Currently, the vaccine has been given both at Motta Hospital and the health center since July last year. Moreover, in February for 10 consecutive days, it was given in campaign. Johnson and Janssen 5179 and pfizer 762 were given both during the campaign and before the campaign, respectively, AstraZeneca one dose 925 and a second dose of 412 population have been vaccinated in the town until March 7 (82, 83). The study was conducted from May 15 to June 15, 2022. The study was conducted from May 15 to June 15, 2022. Although COVID‐19 vaccines had been available in the study area since July 2021 and a community vaccination campaign was conducted in February 2022, this study did not collect information on participants’ reasons for remaining unvaccinated. Therefore, the study assessed intention to receive the vaccine among unvaccinated adults rather than the reasons underlying their vaccination status.

#### 2.1.1 Study Design

A community‐based cross‐sectional study design was employed.

#### 2.1.2 Source Population

All unvaccinated household members in Motta town.

#### 2.1.3 Study Population

All unvaccinated household members in the selected kebeles of Motta town.

#### 2.1.4 Inclusion Criteria

All household members with an age greater than 18 years old were included in the study.

### 2.2 Measurement and Operational Definitions

The instrument comprised sociodemographic (six questions), knowledge assessment (nine questions): It included heard about, sign and symptom, and prevention methods for COVID‐19, and it is vaccine, and questioner was adapted [3, 20, 21] and modified into a local context. Intention to receive COVID‐19 vaccine (4 items, 5‐point Likert scale), attitude measurement (10 items, 5‐point semantic difference scale), and subjective norm (8 items, 5‐point Likert scale), and preceded behavioral control (8 items, 5‐point Likert scale). For the regression analysis, only the direct TPB construct scores (direct attitude, direct subjective norm, and direct PBC) were entered into the multivariable linear regression model. The indirect TPB measures were used to examine construct relationships and were not included in the regression model to avoid conceptual overlap and potential multicollinearity.

#### 2.2.1 Intention to Receive COVID‐19 Vaccine

Community members’ report readiness to receive COVID‐19 vaccine and how much effort they are planning to exert to receive COVID‐19 vaccine was measured by four items using a 5‐point Likert scale, each item was summed up and its score was (4–20), and a higher score indicates high intention to receive COVID‐19 vaccine [22].

#### 2.2.2 Attitude

It is the belief of community members on the usefulness of receiving COVID‐19 vaccine. Directly it was measured with a 6‐point SDS, items were summed to get the score, and higher scores indicate favorable attitude, and indirectly it was measured by five behavioral belief items using a 5‐point Likert scale and multiplied with their corresponding outcome evaluation after reversed negatively coded items according to the theory.

#### 2.2.3 Subjective Norm

Important individuals or groups approve/disapprove community members to receive COVID‐19 vaccine. Directly, it was measured by four items with a 5‐points’ Likert scale, summing up to get the score, and higher score indicates highly influential. Indirect measurement was obtained from items of the normative belief and motivational to comply calculated by computing the product of with their corresponding four each items and summing up to get overall score; higher score shows approval of the behavior according to the theory.

#### 2.2.4 Perceived Behavioral Control

Any environmental or situational factors that inhibit or facilitate community members to receive COVID‐19 vaccine. Directly it was calculated by the overall measure of perceived control over the behavior by five items with 5‐point Likert scale and summed up to get the score, and higher score indicates that the factor was under control and indirectly was measured by multiplication of each four control belief items with their four perceived power items, then summing up product of control belief and perceived power values, and higher score indicates factor will be under control according to the theory.

#### 2.2.5 Knowledge

Study participants were asked nine knowledge questions, and a total score was obtained for each respondent, and summed up to get the score, and a high score shows better knowledge [3].

### 2.3 Sample Size

The sample size for this study was determined using a single population proportion formula by considering 46.6% (*p*) proportion as a definite intention to receive COVID‐19 vaccine from a study done in Ethiopia [15], where the assumption of 95% confidence level and margin of error of 5% and nonresponse rate of 10% was considered. On the basis of these assumptions, the total sample size was calculated using the following formula

$$n={\left(Z\alpha /2\right)}^{2}P\left(1-P\right)/d^{2}$$

where *n* is the minimum possible sample size, *N* is the actual sample size. *Z*α/2 is standard score value for 95% confidence interval level of two‐sided normal distribution = 1.96, and *d* ^2^ is the margin of error = 5%. *P* is the proportion of participant who intend to receive COVID‐19 vaccine = 46.6%. Then minimum possible sample size *n* = (1.96)^2^0.466(1 − 0.466)/(0.05)(0.05):

n=3.8416×0.2488/0.0025=382

Finally, by adding 10% nonresponse rate *N* = 20 and 1.5 design effect by using multistage sampling technique and a total of 630 individual house hold members were involved in the study.

#### 2.3.1 Sampling Technique

Systematic random sampling was applied to select study participants. There are six kebeles in Motta town, of which two kebeles were selected (kebele 01 and 04) by using lottery method. There are a total of 4183 households in the selected two kebeles. Thus, total households were divided by total sample size to find the sampling interval, which was seven. The total number of participants in each kebele was allocated proportionately, *ni* = *n* × *Ni*/*N*. The study households were then chosen using a systematic random sampling procedure every seventh interval. The first participant was randomly chosen by the lottery method, and then every seventh participant was recruited for the study. If more than one eligible participant is found in a household, one is taken by a lottery method to take part in the study.

#### 2.3.2 Data Collection Procedure

Data were conducted using structure interview administered questionnaire and items developed from elicitation study. An open‐ended elicitation study was conducted on 20 individuals from the target population to identify relevant behavioral outcomes, referents, facilitators, and barriers for each particular behavior. Elicitation interviews had 100% response rate, the contents were analyzed by coding related items in to similar constructs, and frequency table was done. Finally, on the basis of frequency of items, TPB constructs were developed. Four and two health extension workers from 01 and 04 kebeles, respectively, were recruited for data collection and one health officer supervisor after 1 day of trading. During data collection, the principal investigator supervised the data collectors and supervisor. The reason why the data were collected by health extension workers is because there was a conflict in the town, and it is difficult to collect data by other people as it is a community‐based study.

### 2.4 Data Management and Analysis

Data were checked for completeness, consistencies and cleaned, coded, and entered into Epi data version 3.1 and then exported into SPSS for windows version 25 for analysis. Descriptive statistics were computed to describe the frequency, percentage, mean, and standard deviation of the study variables. Prior to regression analysis, the assumptions of multiple linear regression were assessed, including the distribution of the intention score, linearity, normality of residuals, homoscedasticity, independence of errors, and multicollinearity. The intention score, obtained by summing four Likert‐scale items (range: 4–20), was treated as a continuous outcome because the composite score was considered appropriate for linear regression after assessment of model assumptions. Bivariable analysis was done to identify candidate variables for multi‐variable linear regression. Multivariable linear regressions were done to predict the association between independent and dependent variables. In the final model, a confidence interval (95%) and a *p* value <0.05 were considered statistically significant.

### 2.5 Data Quality Assurance

The questionnaire was developed in English, then translated into Amharic, and back‐translated into English to check language consistency by language experts with a different person. Being pretested in 5% in Gindewoyine town, 34 km away from the study area, a week before actual data collection period, necessary amendment was made on the questionnaire. Training on the objective of the study, method of data collection, and content of questionnaire was given to supervisor and the data collectors. During data collection days, the principal investigators and supervisors were check completeness. Scale reliability analysis was performed by calculated Cronbach's’ alpha value that was >0.7. A construct validity of item was checked using exploratory factor analysis method. Sampling adequacy was evaluated using the Kaiser–Meyer–Olkin (KMO) measure, and Bartlett's test of sphericity was used to assess the suitability of the data for factor analysis. Factor loadings greater than 0.40 and eigen values greater than 1.0 were considered indicative of acceptable construct validity.

## 3 Results

### 3.1 Sociodemographic Characteristics

A total of 630 community members participated in this study, with a 98.2% response rate. The mean age of the respondents was 32.81 years, with a standard deviation of 9.17, with a range of 18–65 years. About 410 (65.1%) of the participants were orthodox religious followers, and 436 (69.2%) of them were married (see Table 1).

**TABLE 1:** Sociodemographic characteristics of Motta Town Community North West Ethiopia, July 2022 (*N* = 630).

Variables	Category	Frequency	Percentage
Age		Mean = 32.81, SD = 9.174	
Sex	Female	346	54.9
	Male	284	45.1
Religion	Orthodox	410	65.1
	Muslim	220	34.9
Marital status	Married	436	69.2
	Single	141	22.4
	Divorced	53	8.4
Educational status	No formal education	145	23.0
	Primary school (1–8)	128	20.3
	Secondary/preparatory (9–12)	125	19.8
	College/University	232	36.8
Occupational status	Housewife	154	24.4
	Merchant	119	18.9
	Governmental employee	166	26.3
	Non employed	46	7.3
	Student	83	13.2
	Farmer	31	4.9
	Daily laborer	20	3.2
	Others^a^	11	1.7

^a^ Tailors, weavers, and cart drivers.

### 3.2 Knowledge on COVID‐19 and Its Vaccine

All the study participants were heard about COVID‐19. About 592 (94.0%) of the participants were heard about COVID‐19 vaccine. The mean score of knowledge on COVID‐19 was 12.84, with a standard deviation of 4.15. Six hundred twenty four (99.0%) of the participants had ever known prevention methods of COVID‐19 and the respondents considered can be prevented by hand washing 573 (91.0%), keeping physical distance 360 (57.1%), wearing of face mask 551 (87.5%), using sanitizer or alcohol 358 (56.8%), and receiving of COVID‐19 vaccine 534 (84.8%). But, about 186 (29.5%) of the participants responded as no intervention needed to prevent COVID‐19. Majority of the respondents heard about COVID‐19 vaccine from TV or radio 527 (83.7%), and they knew dry cough 415 (65.9%), dyspnea 260 (41.3%), fever 374 (59.4%), and headache 263 (41.7%) as the main symptom of COVID‐19. But, 113 (17.9%) of the participants did not know any sign and symptoms of COVID‐19.

### 3.3 Intention to Receive COVID‐19 Vaccine Among Motta Town Community

**FIGURE 1:**
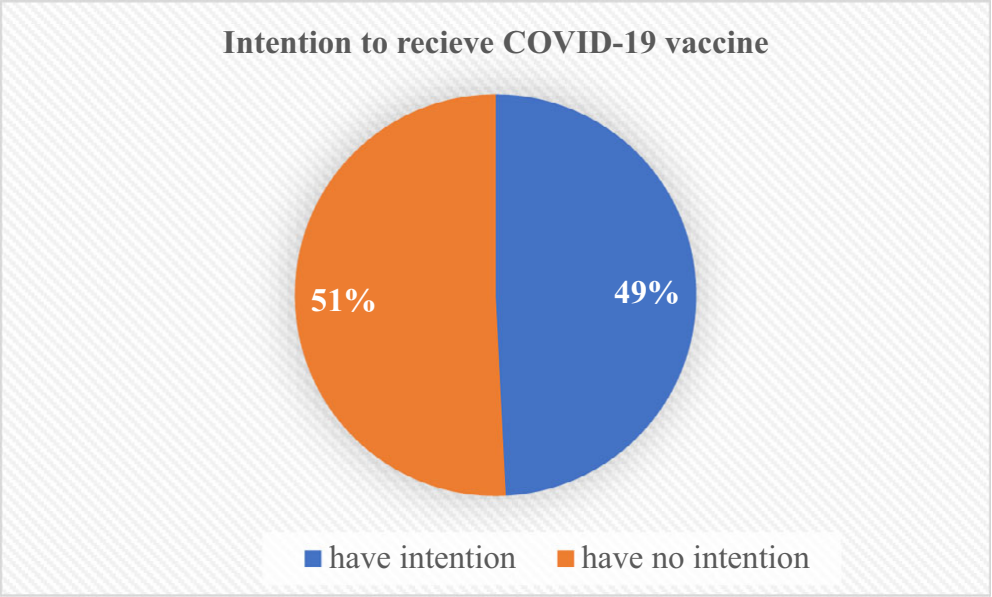
Intention to receive COVID‐19 vaccine among Motta Community North West Ethiopia, July 2022 (*N* = 630).

Intention was measured by four Likert scale questions and computed the total score. A total of 310 (49.2%) with a 95% CI of 45.2%–53.0% of respondents have an intention to receive the COVID‐19 vaccine. The mean score of intention was 12.38, SD ± 5.3 (Figure 1).

### 3.4 Mean Scores of TPB Constructs

For each construct, reliability analysis was done to check the internal consistency of TPB constructs using Cronbach's alpha (*α*). The highest reliability score was for intention (*α* = 0.988), followed by attitude (*α* = 0.978), PBC (*α* = 0.779), and subjective norm (*α* = 0.969).

The mean score of attitude toward intention to receive COVID‐19 vaccine was 58.31 (CI: 56.77–60.04) and SD of 21.31. For subjective norm and PBC, the participants’ scores were 28.48 (CI: 27.00–29.95), SD of 19.389, and PBC, 23.76 (CI: 22.58–24.88), SD of 14.380. Higher scores on the TPB constructs indicate more favorable attitudes toward COVID‐19 vaccination, stronger perceived social support (subjective norms), and greater PBC over receiving the COVID‐19 vaccine (see Table 2).

**TABLE 2:** Descriptive statistics of theory of planned behavior (TPB) components among Motta Community North West Ethiopia, July 2022 (*N* = 630).

TPB construct	Minimum score	Maximum score	Mean	SD
Intention to receive COVID‐19 vaccine	4	20	12.38	5.300
Attitude of receive to COVID‐19 vaccine	17	115	58.31	21.311
Subjective norm of receive to COVID‐19 vaccine	4	100	28.48	19.389
Perceived behavioral control of receive COVID‐19 vaccine	4	76	23.76	14.380

### 3.5 Correlation Among Direct and Indirect Constructs of TPB

There was a strong correlation between direct and indirect constructs of TPB. Direct and indirect attitudes had a strong correlation (*r* = 0.792) (Table 3)

**TABLE 3:** Correlation between direct and indirect construct on theory of planned behavior.

Constructs	DAt	DSN	DPBC	IAt	ISN	IPBC
Dat	1					
DSN	0.675	1				
DPBC	0.601	0.596	1			
IAt	0.792	0.671	0.632	1		
ISN	0.526	0.690	0.405	0.502	1	
IPBC	−0.044	−0.047	−0.078	−0.161	0.142	1

*Note:* Correlation is significant at the 0.01 level (2‐tailed).

Abbreviations: DAt, direct attitude; DPBC, direct perceived behavioral control; DSN, direct subjective norm; IAt, indirect attitude; IPBC, indirect perceived behavioral control; ISN, indirect subjective norm.

### 3.6 Factors Associated With Intention to Receive COVID‐19 Vaccine Among Motta Community

A simple linear regression analysis was conducted to assess the association between intent and other independent variables. Prior to regression analysis, the assumptions of multiple linear regression were assessed. Visual inspection of the intention score and regression residuals indicated no substantial departures from normality or other model assumptions; therefore, multiple linear regression was considered appropriate for the analysis. All variables with a *p* value less than 0.25 in the simple linear regression analysis were entered into multiple linear regression analysis to identify the independent predictors of intention to receive COVID‐19 vaccine. From multiple linear regression analysis, religion (Muslim) was significantly associated with intention to receive COVID‐19 vaccine (*B* = 0.862, *p* < 0.004). Those participants who were Muslin religion followers were 0.862 U increase intended to receive COVID‐19 vaccine. No formal education and primary school education were significantly associated with intention to receive COVID‐19 vaccine (*B* = −1.065, *p* < 0.029) and (*B* = −0.879, *p* < 0.038), respectively.

Individuals who had no formal education (−1.065 U) and those who achieved primary school education (−0.879 U) decreased their intention to receive the vaccine compared to those who were college or university educated. Attitude (*B* = 0.170, *p* < 0.000), subjective norm (*B* = 0.027, *p* < 0.001), and PBC (*B* = 0.029, *p* < 0.004) were statistically significant with intention to receive COVID‐19 vaccine.

For a unit positive change in attitude towards COVID‐19 vaccine, intention to receive COVID‐19 vaccine will be increased by 0.170 U, and for a positive unit change in individuals that approve of receiving COVID‐19 vaccine, intention to receive COVID‐19 vaccine will be increased by 0.027 U provided that other variables are kept constant. For a positive unit change in perceived control of beliefs on environmental/situational facilitators to receive COVID‐19 vaccine; intention will be increased by 0.029 U. This model explains 59.8% of the variance (adjusted *R* squared = 0.598). Only the direct TPB construct scores were included as predictors in the multivariable linear regression model. Indirect TPB measures were examined separately through correlation analysis and were not entered into the regression model (see Table 4).

**TABLE 4:** Factors associated with intention to receive COVID‐19 vaccine among Motta Community North West Ethiopia July 2022.

		Unstandardized coefficients		Standardized coefficients			95% confidence interval for *B*	
Variable		*B*	Std. error	Beta	*t*	*p* value	Lower bound	Upper bound
Age		−0.024	0.017	−0.042	−1.395	0.163	−0.058	0.010
Religion	Muslim Orthodox(ref.)	0.862	0.297	0.078	2.902	0.004^a^	0.279	1.445
Marital status	Single	−0.503	0.358	−0.040	−1.407	0.160	−1.206	0.199
	Married(ref.)							
Educational status	No formal education	−1.065	0.486	−0.085	−2.190	0.029^a^	−2.020	−0.110
	College/University educated(ref.)							
	Primary school educated (1–8)	−0.879	0.424	−0.067	−2.075	0.038^a^	−1.711	−0.047
	Secondary/preparatory school educated	0.173	0.398	0.013	0.435	0.664	−0.608	0.954
Occupation	Housewife	0.141	0.384	0.011	0.368	0.713	−0.613	0.896
	Government employee (ref.)							
	Non‐employee	−0.557	0.544	−0.027	−1.023	0.307	−1.625	0.512
	Government employee (ref.)							
	Farmer	−0.169	0.709	−0.007	−0.238	0.812	−1.562	1.224
	Government employee(ref.)							
Knowledge		−0.036	0.038	−0.028	−0.955	0.340	−0.111	0.038
Attitude		0.170	0.008	0.683	22.17	0.000^a^	0.155	0.185
Subjective norm		0.027	0.008	0.100	3.280	0.001^a^	0.011	0.044
Perceived behavioral control		0.029	0.010	0.078	2.923	0.004^a^	0.009	0.048

Abbreviations: PBC, perceived behavioral control; ref., reference.

^a^Shows significant variable at *p* value <0.05, adjusted *R* square = 59.8%.

### 3.7 Exploratory Factor Analysis

Exploratory factor analysis was conducted to check the validity of the measurement using factor loadings and eigen values on intention, attitude, subjective norm, and PBC. The analysis results of each item in all construct factor loadings are greater than 0.4, and the eigen value is greater than 1. All KMO values are greater than 0.7 and Bartlett's test of Sphericity is significant (see Table 5).

**TABLE 5:** Exploratory factor analysis for the constructs of theory of planned behavior of intention to receive COVID‐19 vaccine among Community Motta town North West Ethiopia July 2022.

Construct	Items	Definition	Factor loading	Eigen value
Intention	Q301	Question 301	0.987	3.865
	Q302	Question302	0.983	
	Q303	Question303	0.982	
	Q304	Question304	0.980	
Attitude	Q31116	Question311^*^316	0.951	3.306
	Q31217	Question312^*^317	0.767	
	Q31318	Question313^*^318	0.811	
	Q31419	Question314^*^319	0.566	
	Q31520	Question315^*^320	0.915	
Subjective norm	Q32529	Question25^*^329	0.910	2.682
	Q32630	Question326^*^330	0.878	
	Q32731	Question327^*^331	0.850	
	Q32832	Question328^*^332	0.601	
Perceived behavioral control	Q33842	Question338^*^342	0.856	2.573
	Q33943	Question339^*^343	0.840	
	Q34044	Question340^*^344	0.776	
	Q34145	Question341^*^345	0.736	

## 4 Discussion

In the present study, intention to receive COVID‐19 and associated factors among the community of Motta Town using TPB were assessed. Only about half of the unvaccinated participants intended to receive the COVID‐19 vaccine, indicating that a substantial proportion remained unwilling or uncertain despite vaccine availability. This finding has important public health implications because achieving high vaccination coverage depends not only on vaccine availability but also on public willingness to accept vaccination. The relatively low level of intention suggests that interventions should address both behavioral determinants and contextual barriers that influence vaccine uptake.

Because the study was conducted after vaccine rollout had begun, remaining unvaccinated may have reflected a range of circumstances, including vaccine hesitancy, delayed acceptance, logistical barriers, or missed opportunities. As these reasons were not directly measured, the observed intention should not be interpreted solely as evidence of vaccine hesitancy. This was similar to a study conducted in Southern Ethiopia that 48.4% [23, 24]. But lower than the study done on Gondar teachers, which was 54.8% [22]. The variation might be due to knowledge differences.

This study is lower than studies done in Nigeria (59.8%) [25], the Democratic Republic of the Congo (55.9%) [21], and Mozambique (71.4%) [26, 27], which was also lower than the results of studies conducted in Italy (94.73%), Canada (79.85%), China (83.8%), Japan (65.7%), Vietnam (76.10%), Greece (57.7%), France (77.6%), and Portugal (63.3%) [28, 29, 30]. The variation of results might be due to sociodemographic differences like perception of risk and susceptibility to COVID‐19 influenced vaccine intention and areas where highly COVID‐19 pandemic zones [24, 31, 32].

The result of this study showed that Muslims were significantly associated with the intention to receive COVID‐19 vaccine. Being of the Muslim religion increased the intention to receive the COVID‐19 vaccine by 0.879 compared to orthodox followers, which is in line with studies done in Gondar and an online survey in Ethiopia [22, 29, 33]. The possible reasons might be related to a low perceived risk of becoming infected or vaccine resistance due to religious beliefs and religion objection contributing to lower vaccine uptake [34].

According to this study, no formal education and primary education were significantly associated with intention to receive COVID‐19 vaccine. Participants who were not formal educated had a −1.065 U decrease in intention to receive COVID‐19 vaccine when compared to those who were college or university educated, and those who were primary school educated had a −0.879 U decrease in intention to receive the COVID‐19 vaccine when compared to those who were college or university educated, which was similar to studies done in the United States and Australia [35, 36]. This might be due to educated individuals being aware of the benefits of the COVID‐19 vaccine. This is in contrary study done in Gondar [22]. The possible reason might be due to differences in study design, which was facility‐based, whereas our study was community‐based. Although educational status was significantly associated with vaccination intention, this relationship should not be interpreted solely as reflecting differences in knowledge. Educational attainment may also be linked to which can influence individuals’ ability to access, understand, evaluate, and use health information when making vaccination decisions. Recent evidence indicates that lower educational attainment is associated with greater vaccine hesitancy, whereas higher levels of general and communicative health literacy are associated with lower vaccine hesitancy [37]. This provides a plausible mechanism for our finding that participants with no formal education and those with primary education had lower vaccination intention than those with college/university education. Individuals with lower educational attainment may have fewer opportunities to develop the skills needed to critically evaluate vaccine information, understand vaccine benefits and risks, and navigate vaccination services. However, because health literacy was not directly measured in the present study, this interpretation should be considered a plausible explanatory pathway rather than a demonstrated causal mechanism. Other factors associated with educational attainment, including socioeconomic circumstances, healthcare access, and trust in health institutions, may also contribute to differences in vaccination intention [37].

In the present study, from the TPB constructs, attitude, subjective norm, and PBC had a significant and positive association with the intention to receive COVID‐19 vaccine. This study showed that attitude was found to be a statistically significant predictor of intention to receive the COVID‐19 vaccine. When attitude increased a unit, intention to receive COVID‐19 vaccine increased by 0.170 U, which was congruent to studies done in southern eastern Ethiopia and Northern Ireland [38, 39]. The possible reason might be that an individual who had a positive attitude toward COVID‐19 vaccine has an increased intention to receive the vaccine [23].

Subjective norm was significantly associated with intention to receive COVID‐19 vaccine, a unit increase subjective norm result increase intention to receive COVID‐19 vaccine by 0.027 U, which was in line with study done south Iran and Saudi Arabia [40, 41]. This might be an individual who had a positive subjective norm and an intention to receive the COVID‐19 vaccine, and those who favor social pressure and have a positive normative belief are more likely to receive the COVID‐19 vaccine [41].

PBC was significantly associated to intention to receive COVID‐19 vaccine, a unit increase PBC result increase intention to receive COVID‐19 vaccine by 0.029 U, which was congruent with studies done in Nigeria and Northern India [42, 43]. The possible reason was that due to fear of being a risk, everyone had high motivation to receive COVID‐19 vaccine. This is contrary to research done in America [44]. This might be due to lower risk perception and fear as a negative emotion.

Although the TPB provides a useful framework for understanding individual‐level determinants of vaccination intention, these factors operate within broader social and structural contexts. Access to vaccination services, institutional trust, health literacy, and socioeconomic conditions may also influence vaccination decisions. Therefore, behavioral interventions should be complemented by strategies that address structural barriers to improve vaccine uptake.

### 4.1 Limitation of the Study

There might be socially desirability biases and interventions that made them were told of all the details of the study before giving their consent to the participants. The study did not assess why participants remained unvaccinated despite vaccine availability. Consequently, it was not possible to distinguish between vaccine hesitancy, access barriers, previous refusal, delayed acceptance, or other contextual factors. This should be considered when interpreting the findings. Although a multistage sampling technique was employed, the regression analysis did not explicitly account for clustering at the kebele level. This may have resulted in underestimated standard errors and overly precise estimates; therefore, the precision and statistical significance of the associations should be interpreted cautiously. In addition, this study was conducted during May–June 2022, a specific stage of the COVID‐19 pandemic when vaccine availability, vaccination campaigns, and community risk perceptions were evolving. Therefore, the findings reflect vaccination intention at that particular time and may not be generalizable to other phases of the pandemic or to changing epidemiological contexts.

## 5 Conclusions

Approximately half (49.2%) of the unvaccinated community members intended to receive the COVID‐19 vaccine. Religion, educational status, attitude, subjective norm, and PBC were significant predictors of vaccination intention. As the study was conducted after COVID‐19 vaccine rollout, remaining unvaccinated may have reflected not only vaccine hesitancy but also access‐related or contextual barriers that were not assessed. Public health interventions should strengthen positive attitudes, supportive social norms, and individuals’ confidence in accessing vaccination while also addressing structural barriers through community engagement, targeted health communication, and improved access to vaccination services.

## Author Contributions

**Alemnew Getachew**, **Nestanet Fentahun**, **Gebeyehu Lakew**, and **Yosef Wasihun** participated in the conception and design of the study. **Alemnew Getachew** carried out data collection. **Alemnew Getachew**, **Nestanet Fentahun**, and **Yosef Wasihun** participated in the data analysis and interpretation. **Nestanet Fentahun** and **Yosef Wasihun** drafted the manuscript. **Alemnew Getachew**, **Nestanet Fentahun**, **Yosef Wasihun**, and **Gebeyehu Lakew** reviewed and edited the manuscript. All authors read and approved the final manuscript.

## Funding

The authors have nothing to report.

## Ethics Statement

Ethical approval was obtained from the Institutional Review Board (IRB) of Bahir Dar University, College of Medicine and Health Sciences with reference number 440/2022. The IRB of Bahir Dar University decided and approved that verbal informed consent obtained from each study participant could be enough to be ethically assured of the research process. A permission letter was taken from Motta town health office and permission to undertake the study was obtained at all levels.

## Consent

All the study participants were informed about the purpose of the study. The respondents were informed of the right to refuse participation or terminate their involvement at any point during the study. Information provided by each respondent was kept confidential.

## Conflicts of Interest

The authors declare no conflicts of interest.

## Data Availability

The data that support the findings of this study are available on request from the corresponding author. The data are not publicly available due to privacy or ethical restrictions.
